# Response of Bacterial Communities to Different Detritus Compositions in Arctic Deep-Sea Sediments

**DOI:** 10.3389/fmicb.2017.00266

**Published:** 2017-02-24

**Authors:** Katy Hoffmann, Christiane Hassenrück, Verena Salman-Carvalho, Moritz Holtappels, Christina Bienhold

**Affiliations:** ^1^HGF-MPG Joint Research Group for Deep Sea Ecology and Technology, Max Planck Institute for Marine MicrobiologyBremen, Germany; ^2^Biosciences, HGF-MPG Joint Research Group for Deep Sea Ecology and Technology, Alfred-Wegener-Institut Helmholtz-Zentrum für Polar- und MeeresforschungBremerhaven, Germany; ^3^Biosciences, Bentho-Pelagic Processes, Alfred-Wegener-Institut Helmholtz-Zentrum für Polar- und MeeresforschungBremerhaven, Germany

**Keywords:** climate change, surface sediment, food pulse experiment, opportunistic bacteria, 16S Illumina sequencing, Arctic algae

## Abstract

Benthic deep-sea communities are largely dependent on particle flux from surface waters. In the Arctic Ocean, environmental changes occur more rapidly than in other ocean regions, and have major effects on the export of organic matter to the deep sea. Because bacteria constitute the majority of deep-sea benthic biomass and influence global element cycles, it is important to better understand how changes in organic matter input will affect bacterial communities at the Arctic seafloor. In a multidisciplinary *ex situ* experiment, benthic bacterial deep-sea communities from the Long-Term Ecological Research Observatory HAUSGARTEN were supplemented with different types of habitat-related detritus (chitin, Arctic algae) and incubated for 23 days under *in situ* conditions. Chitin addition caused strong changes in community activity, while community structure remained similar to unfed control incubations. In contrast, the addition of phytodetritus resulted in strong changes in community composition, accompanied by increased community activity, indicating the need for adaptation in these treatments. High-throughput sequencing of the 16S rRNA gene and 16S rRNA revealed distinct taxonomic groups of potentially fast-growing, opportunistic bacteria in the different detritus treatments. Compared to the unfed control, *Colwelliaceae, Psychromonadaceae*, and *Oceanospirillaceae* increased in relative abundance in the chitin treatment, whereas *Flavobacteriaceae, Marinilabiaceae*, and *Pseudoalteromonadaceae* increased in the phytodetritus treatments. Hence, these groups may constitute indicator taxa for the different organic matter sources at this study site. In summary, differences in community structure and in the uptake and remineralization of carbon in the different treatments suggest an effect of organic matter quality on bacterial diversity as well as on carbon turnover at the seafloor, an important feedback mechanism to be considered in future climate change scenarios.

## Introduction

Deep-sea sediments below 200 m water depth cover approximately 65% of the Earth’s surface. In this environment, particle flux from surface waters is the main source of energy and carbon to benthic communities, with only 1–5% of the exported organic material arriving at the seafloor (Klages et al., 2003; Jørgensen and Boetius, 2007). A small fraction (1–2%) of the arriving carbon is remineralized in surface sediments within a few days, while much of the remainder gets buried, turning the seabed into the globally most important long-term sink for carbon (Jørgensen and Boetius, 2007). Bacterial communities may constitute up to 90% of total benthic biomass in the deep sea (Rowe, 1991; Pfannkuche, 1992), serving as essential catalysts in carbon and nutrient cycling (Jørgensen and Boetius, 2007). The availability of organic matter has been identified as a major driver structuring deep-sea communities, including bacterial communities (e.g., Zinger et al., 2011; Bienhold et al., 2012, 2016). Assessing the response of bacterial communities to changing carbon input is therefore important to understand matter fluxes in the deep sea, especially in the context of global climate change.

In this regard, the Arctic Ocean plays a prominent role because of the rapid environmental changes occurring in this region. As a consequence of increasing surface water temperatures and sea-ice retreat, the composition and amount of Arctic phytoplankton and sinking organic matter is currently undergoing major changes (Michel et al., 2006; Arrigo et al., 2008; Bauerfeind et al., 2009). In extreme cases, the sudden, massive export of fresh sea-ice algae to the deep-sea floor in the Central Arctic resulted in local hypoxic conditions beneath such algal patches (Boetius et al., 2013). Despite these changes in the quality and quantity of organic matter exported to the deep-sea benthos, little is known about the effects on benthic community structure and functioning. Therefore, the major objective of this study was to evaluate the response of Arctic benthic bacterial deep-sea communities to different organic matter sources.

Methodologically, the retrieval of deep-sea sediment samples, and the large diversity of benthic bacterial communities are two major challenges in this area of research. In contrast to logistically challenging *in situ* experiments, *ex situ* studies (Boetius and Lochte, 1994; Boetius and Damm, 1998; Mayor et al., 2012) allow for comparisons between different treatments under controlled conditions, including sample replication. Previous studies have provided simple nutrient sources to investigate the response of bacterial communities to organic matter input (Jannasch et al., 1973; Jannasch and Wirsen, 1982; Deming and Colwell, 1985). Few have also used more complex food sources, such as chitin and whole algae (Deming, 1985; Moodley et al., 2002; Kanzog et al., 2009), which represent more environmentally relevant sources of organic matter. Results indicated that bacterial communities respond to the deposition of organic matter within a few days, as recorded by increases in the activity of extracellular enzymes, bacterial biomass, and community oxygen consumption (Turley and Lochte, 1990; Witte et al., 2003b). However, only more recent studies included analyses of whole bacterial community structure, or single taxonomic groups by applying fingerprinting methods (Yanagibayashi et al., 1999; Kanzog et al., 2009), cloning, and sanger sequencing (Toffin et al., 2004; Parkes et al., 2009; Gärtner et al., 2011). Other studies that sampled along natural environmental gradients of organic matter availability have identified potential opportunistic groups, e.g., *Alteromonadaceae, Psychromonadaceae*, and *Flavobacteriaceae* which were positively related with organic matter availability (Bienhold et al., 2012; Jacob et al., 2013). But, experimental studies still lacked combined measurements of community function and high-resolution taxonomic community structure in response to the addition of natural complex food sources.

Here, we used deep-sea surface sediments from the Arctic Ocean Long-Term Ecological Research Observatory (LTER) HAUSGARTEN located in the Fram Strait (Soltwedel et al., 2015) to perform experimental carbon additions, and monitor the response of benthic bacterial communities. The particulate organic carbon flux at this well-studied site is generally less than 30 mg C m^-2^ d^-1^ (∼1 mg C mL^-1^ yr^-1^) at 200–300 m water depth, and is mainly composed of fecal pellets, calcium carbonate, refractory particulate organic carbon, and biogenic particulate silica (Bauerfeind et al., 2009; Lalande et al., 2013). Since 2005, strong declines in diatom numbers, such as *Thalassiosira weissflogii*, have led to shifts in phytoplankton community composition. At the same time, a dominance of coccolithophores, especially *Emiliania huxleyi*, as well as the prymnesiophyte *Phaeocystis* was observed in the sinking material (Bauerfeind et al., 2009; Soltwedel et al., 2015), but effects on benthic communities remain unknown.

In this study, deep-sea sediment slurries were amended with different types of organic matter, including four different naturally occurring Arctic algae species, and chitin as the most abundant biopolymer in the oceans, e.g., as a main component of fecal pellets. Incubations were performed at *in situ* temperature and atmospheric pressure, with parallel *in situ* pressure incubations as controls. After 23 days, changes in key community functions were assessed along with changes in bacterial taxonomic composition. In contrast to natural environmental gradients that may have persisted over extended periods of time, the present study has a short experimental duration and specifically targets fast-growing bacterial groups. We hypothesized (i) that the input of different particulate organic carbon sources to deep-sea sediments differentially affects microbial degradation rates and growth, (ii) that community structure varies with organic matter types, and (iii) that we can identify rapidly responding, i.e., fast-growing, potentially polymer-degrading bacterial groups, representing an opportunistic part of the sediment microbiome.

## Materials and Methods

### Sediment Sampling

Surface sediments were sampled at the central station of the LTER HAUSGARTEN, located in the Fram Strait west of Spitsbergen (79° 03.86′ N, 4° 10.85′ E; 2,470 m water depth). A TV-guided multiple corer (MUC; Barnett et al., 1984) was used for retrieving undisturbed sediments during RV Polarstern cruise PS93.2 (PS93/050-5 and -6; Soltwedel, 2015). Surface sediments were fully oxic (300 μmol L^-1^ O_2_) and consisted of fine clays (Kanzog et al., 2008). Immediately after sediment retrieval, a control sample ‘day 0’, representing the *in situ* bacterial community structure, was taken and frozen at –80°C for RNA and at –20°C for DNA extractions.

For the feeding experiment, the first two centimeters of ten cores were combined into one sterile glass bottle and transferred into a cold room (0°C, *in situ* temperature). The sediment was 3.5-fold diluted with sterile-filtered and air saturated bottom water (35 PSU; 0°C), in order to supply enough oxygen for at least one community duplication. The well-mixed slurry was divided into six sterile 1 L glass bottles. One bottle with slurry remained as unfed control. The other five bottles were amended with substrates to final concentrations of 0.2 mg organic carbon per mL undiluted sediment, which corresponds to 16.6 mmol L^-1^ organic carbon, and is comparable to an annual input of particulate organic carbon at this site (Bauerfeind et al., 2009; Kanzog and Ramette, 2009; Lalande et al., 2013). All treatments were divided into ten replicates of 50 mL each, transferred and sealed into sterile, gas-permeable polyethylene (PE) bags with no headspace or air bubbles.

### Incubation

Slurry incubations were started within 24 h after retrieval of the samples, using pressure vessels described in Boetius and Lochte (1994). Ten pressure vessels were filled with fully oxygenated sterile-filtered bottom water, and six PE bags were added to each of them (one replicate bag of each of the five treatments, and one control bag). Five of these pressure vessels were incubated at atmospheric pressure (1 atm), the other five at *in situ* pressure (250 atm). Pressure was applied as hydrostatic (water) pressure, using a mechanical pump. The PE bag material was oxygen permeable, so that oxygen diffusion into the bags prevented the slurries from becoming anoxic (Supplementary Table S1; Supplementary Data). All incubators were stored at 0°C in the dark for 23 days.

### Food Sources Used in this Study

We selected five different, naturally occurring, complex substrates to test functional and structural responses of Arctic deep-sea bacterial communities. These were: chitin (CHI), *Thalassiosira weissflogii* (TWEI), *Emiliania huxleyi* (EHUX), *Bacillaria sp*. (BCLA), and *Melosira arctica* (MARC).

Chitin is one of the main components of, e.g., fecal pellets, exoskeletons of worms, arthropods, and mollusks, and thus constitutes a key source of carbon and nitrogen to deep-sea life (Wakeham and Lee, 1993). Here, we used a commercially available product (flakes from shrimp shells, Sigma–Aldrich, Germany). As more complex detritus we added different algae: the centric diatom TWEI, representing a major fraction of sinking particulate organic matter at HAUSGARTEN observatory, as well as the coccolithopore EHUX that has been reported to migrate from the Atlantic towards the Arctic (Bauerfeind et al., 2009; Winter et al., 2014). Furthermore, we provided detritus of the sea-ice algae BCLA (Stecher et al., 2015), a pennate diatom, and MARC, a centric diatom. As a consequence of sea-ice melting, these sea-ice algae can cause massive sedimentation events that strongly impact local sediment turnover rates (McMahon et al., 2006; Boetius et al., 2013). In reference to the incubations, the term phytodetritus includes all algae used (EHUX, TWEI, BCLA and MARC), of which EHUX and TWEI belong to phytoplankton, and BCLA and MARC to the sea-ice algae.

### Algal Cultivation, Sterilization, and Tests Prior to Usage

TWEI, EHUX, and BCLA were collected during several Arctic cruises, and were cultivated in F/2-medium (Guillard and Ryther, 1962; Guillard, 1975). Algal mats dominated by MARC (but also containing few other species of pennate diatoms and *Chaetoceros* sp.) were collected directly from the ice during Polarstern cruise PS86 in July 2014 (Boetius, 2015) and frozen at –20°C.

The phytoplankton species (TWEI and EHUX) were incubated in 2.5 L F/2 medium at 18°C and 15 μE using 5 L beakers, and were resuspended twice a week by gentle shaking. TWEI and EHUX were transferred into new media every 10 days. 100 mL of the 2.5 L culture were used to inoculate fresh medium, while the remainder was centrifuged at 1811 × *g*, and the pellet was frozen at –20°C. BCLA was grown at 0°C and 10 μE in 400 mL medium in a 1 L beaker. Due to slower growth, it was transferred and harvested once a month as described above.

For inactivating algal-attached bacteria, we sterilized the algae with microwave radiation at 600 W for three minutes before adding them to the slurry. Sterilization may alter organic matter lability, as has been shown for the process of autoclaving (Turley and Lochte, 1990; Wolf and Skipper, 1994). This may, to some extent, cause changes similar to the natural aging of organic matter, but was not quantified in more detail here. This treatment also led to a disintegration and homogenization of the algae cultures. The effective sterilization was tested by measurements of enzyme activity, oxygen uptake, bacterial cell numbers, and bacterial genetic diversity, which were all negative or without significant increase or change. The carbon and nitrogen content of the different food sources were determined according to Grasshoff et al. (1983), using a Fisons element analyzer type NA 1500 (Fisons plc, UK). C:N ratios were 8:1 for CHI, 10:1 for TWEI, 11:1 for EHUX, 12:1 for BCLA and 23:1 for MARC.

### Acridine Orange Direct Cell Counts (AODC)

To determine prokaryotic cell numbers, 1 mL sediment slurry was fixed with sterile filtered formalin/seawater at a final concentration of 2% and stored at 4°C. Each sample was filtered on a 0.2 μm polycarbonate filter, stained with acridine orange as DNA dye (0.01% final concentration), and counted using an epifluorescence microscope (Axiophot II Imaging, Zeiss, Jena, Germany). For each sample, 30 random grids from two replicate filters (technical replicates) of three biological replicates, representing three out of five replicate PE-bags per treatment, were counted (Meyer-Reil, 1983). Bacterial biomass was estimated using a conversion factor of 3 × 10^-13^ g C μm^-3^ biovolume (Børsheim et al., 1990), and assuming an average cell volume of 0.07 μm^3^ (Boetius and Lochte, 1996).

### Potential Extracellular Enzymatic Activity (EEA)

By measuring the potential hydrolytic activity of two abundant enzymes, N-acetylglucosaminidase (chitobiase) and beta-glucosidase, in a modification of the method by Hoppe (1983) we tested, which of the supplied macromolecular organic substrates induced a production of extracellular enzymes in the community. Two 11 mL subsamples were taken from each of the five replicate PE-bags, and mixed with the methylumbelliferon (MUF)-labeled artificial substrates 4-methylumbelliferyl ß-D-glucopyranoside (MUF-beta-glucosidase) and 4-methylumbelliferyl-*N*-acetyl-ß-D glucosaminide (MUF-chitobiase), at saturation levels (100 μmol L^-1^) according to the method described in Boetius and Lochte (1994; Supplementary Table S2). The assay was run at 0°C for three hours at atmospheric pressure. We measured the release of the fluorochrome (MUF) after one and three hours, using a RF-5300 spectrofluorophotometer (Shimadzu Scientific, USA; emission: 455 nm, excitation: 365 nm). Fluorescent activity was calibrated with MUF standards ranging between 0 and 6 nmol mL^-1^. Extracellular enzymatic activity (EEA) was then calculated per volume of sediment and time (μmol mL^-1^ d^-1^). Since substrate concentrations are at saturation level within the incubation time, enzyme activities represent maximum velocities (Vmax). Enzymatic activity measurements may be confounded by competitive inhibition between the fluorogenic substrate and the added carbon (Arnosti, 2011). However, previous studies have reported an induction of enzyme production for beta-glucosidase and chitobiase by their respective substrates (Boetius and Lochte, 1994; Boetius and Lochte, 1996).

### Oxygen Uptake

Oxygen concentrations were measured at the end of the incubation in each of the five replicate PE-bags per treatment as an indicator of community activity over the duration of the experiment. Optodes were connected to the FireStingO_2_ fiber-optic oxygen meter (both obtained from Pyro Science GmbH, Germany), and calibrated at *in situ* temperatures by a two-point calibration using air-purged seawater and oxygen-free seawater by adding sodiumhydrogensulfite (Sigma Aldrich, Germany). The measuring accuracy was between 0.2% at 20% oxygen and 0.01% at 1% oxygen (FireSting O_2_, Fiber-optic Oxygen Meter manual, Pyro Science GmbH, Germany). The PE-bags were gently shaken to mix the slurries and punctured immediately after retrieval from the incubator with the needle-protected optode fiber. Oxygen concentrations were measured in the center of the slurries. Because the PE-bags were oxygen-permeable, the measured oxygen concentration at the end of the 23 days of incubation was in steady state, i.e., the microbial respiration in the bag was balanced by the diffusive transport of oxygen across the PE-foil. From previous tests, an oxygen diffusion coefficient of 2.65 × 10^-12^ m^-2^ s^-1^ was determined and in combination with the oxygen difference across the PE-foil, the bulk flux and thus the respiration rate in each of the bags were determined (Supplementary Data; Supplementary Table S1; Supplementary Figure S1).

### DNA/RNA Extraction and cDNA Generation

The analysis of 16S rRNA-genes (16S rDNA) reveals all taxa present in a given sample, while the analysis of the 16S rRNA identifies those taxa actively transcribing RNA under a given condition. From the latter, the metabolically active bacterial community can be inferred, and community shifts may be more easily detected (Moeseneder et al., 2005; Gaidos et al., 2011).

For DNA extraction, 7 mL slurry of three replicate PE-bags per treatment were sampled, centrifuged (3 min at 3000 rpm to remove the overlaying water) and stored at –20°C. Total DNA was extracted from 0.5 g sediment with the UltraClean Soil DNA Isolation Kit (MoBio Laboratories Inc., Carlsbad, CA, USA), and quantified using a microplate spectrometer (Infinite^®^200 PRO NanoQuant, TECAN Ltd, Switzerland). For assessing the active fraction of the bacterial community, 20 mL slurry of one replicate per treatment were sampled, immediately frozen in liquid nitrogen and stored at –80°C. Total RNA was extracted from 5 g sediment with the MoBio PowerSoil RNA extraction Kit (MoBio Laboratories Inc., Carlsbad, CA, USA). After DNAse digestion and purification using the RNeasy MinElute Cleanup Kit (QIAGEN, Germany), the purity and quantity were determined by electrophoresis using the 2100 Bioanalyzer (Agilent Technologies, Inc., Santa Clara, CA, USA). The extracts were translated into cDNA using the qScriptTM cDNA SuperMix Kit (Quanta Biosciences, Gaithersburg, MD, USA).

### Amplicon Sequencing and Sequence Processing

For 16S cDNA amplicon library preparation, the standard instructions of the 16S Metagenomic Sequencing Library Preparation protocol (Illumina, Inc., San Diego, CA, USA) were followed. The hypervariable V3–V4 region of the bacterial 16S cDNA and rDNA was sequenced using the bacterial primers S-D-Bact-0341-b-S-17 (5′-CCTACGGGNGGCWGCAG-3′) and S-D-Bact-0785-a-A-21 (5′-GACTACHVGGGTATCTAATCC-3′; Klindworth et al., 2013). Sequences were obtained on the Illumina MiSeq platform in a 2 × 300 bp paired-end run as well as in a 2 × 250 bp paired-end run on the Illumina HiSeq platform (CeBiTec Bielefeld, Germany). Raw paired-end reads were primer-trimmed using cutadapt (Martin, 2011). For quality trimming, a sliding window of four bases and a minimum average quality of 15 was applied in trimmomatic v0.32 (Bolger et al., 2014), and the reads were merged using PEAR v0.9.5 (Zhang et al., 2014). Clustering into OTUs was done with the swarm algorithm using default parameters (v2.0; Mahé et al., 2014). One representative sequence per OTU was taxonomically classified with SINA (SILVA Incremental Aligner; v1.2.11; Silva reference database release 123) at a minimum alignment similarity of 0.9, and a last common ancestor consensus of 0.7 (Pruesse et al., 2012). OTUs that were classified as chloroplasts, mitochondria, *Archaea*, and those not classified at the domain level were excluded from further analyses, as well as OTUs that occurred with only a single sequence in the whole dataset.

### Statistical Analysis

Enzyme activity, cell abundances, and respiration rates based on oxygen measurements were calculated per volume of undiluted sediment. Differences between treatments were calculated based on ANOVA (analysis of variance) with Tukey HSD *post hoc* tests at a significance threshold of 0.05 (Hothorn et al., 2008). For cell counts, where technical replicates were available, mixed model ANOVA and the implementation of Tukey HSD in the R package multcomp were used. EEA and AODC data were square-root transformed to meet the assumptions of ANOVA.

Observed richness (number of OTUs) and evenness (inverse Simpson index) of the communities, were calculated via repeated random subsampling to the minimum library size of the amplicon data set (i.e., 26,712 sequences). The change in community structure (beta-diversity) between samples was assessed by calculating Bray–Curtis dissimilarities from relative OTU abundances [%], to produce non-metric multidimensional scaling (NMDS) plots, and to visualize community similarity between the 16S rRNA and rDNA datasets as well as between the different treatments.

The R-package ALDEx2 (ANOVA Like Differential Expression) was used to identify differentially abundant OTUs and families between treatments in the rDNA data set. Only OTUs that were present in two out of three replicates were used for the ALDEx2 analysis. Prior to analysis, proportional 16S rDNA based OTU abundances were centered log ratio (clr-) transformed using the aldex.clr function in R with 128 Dirichlet instances (Fernandes et al., 2014). Taxa were classified as differentially abundant at an adjusted parametric significant threshold of 0.05 (Benjamini and Hochberg, 1995), and at an unadjusted, non-parametric significance threshold of 0.05. Furthermore, the pairwise average difference and the effect size between the treatments at day 23 compared to the start of the incubation were calculated in the rDNA data set (Fernandes et al., 2014). Taxa were identified as responding to the treatments at an effect size of more than 4 and an average difference between treatments of more than log_2_2 or less than log_2_0.5, i.e., one duplication or decrease by half in relative abundance. Since only one sample per treatment was available for the rRNA data set, no statistical analyses could be performed. Here, only abundant taxa with a relative sequence abundance of at least 1% (on OTU level) in at least one sample were screened for responses to the carbon treatments, and only taxa exhibiting at least one duplication or a decrease by half after 23 days are reported. On family level, only differentially abundant taxa with relative sequence abundances above 2% in at least one sample will be discussed.

All statistical analyses were conducted in R using the core distribution (version 3.3.0; R Development Core Team, 2014) and the following packages: vegan (Oksanen et al., 2015), ALDEx2 (Fernandes et al., 2014), nlme (Pinheiro et al., 2016), and multcomp (Hothorn et al., 2008).

### Data Accession Numbers

Data are accessible via the Data Publisher for Earth & Environmental Science PANGAEA^1^ (Hoffmann et al., 2016). Raw paired-end sequence, primer-trimmed reads are available on ENA Accession Number: PRJEB17614. The data were archived using the brokerage service of GFBio (Diepenbroek et al., 2014).

## Results

To assess the response of an Arctic benthic bacterial community to different detritus additions, we determined bacterial cell numbers, estimated biomass, and measured enzymatic activities of two extracellular enzymes, as well as oxygen uptake. Variations in total (16S rDNA) and metabolically active (16S rRNA) bacterial community structure were assessed with 16S tag sequencing of the V3-V4 region. The observed effects represent net community changes, as sediments were unsieved, allowing for bacterial grazing by, e.g., protists, and viral pressure. All data presented below are based on incubations conducted at atmospheric pressure (1 atm). General patterns were comparable between incubations at atmospheric and *in situ* pressure conditions at 250 atm. Corresponding results are shown in the Supplementary Material (Supplementary Figures S2–S6 and Supplementary Tables S3 and S4). We present here comparisons between the initial sediment community at day 0 and the community incubated for 23 days with or without (unfed control) carbon amendments, if not stated otherwise.

### Cell Biomass

**FIGURE 1:**
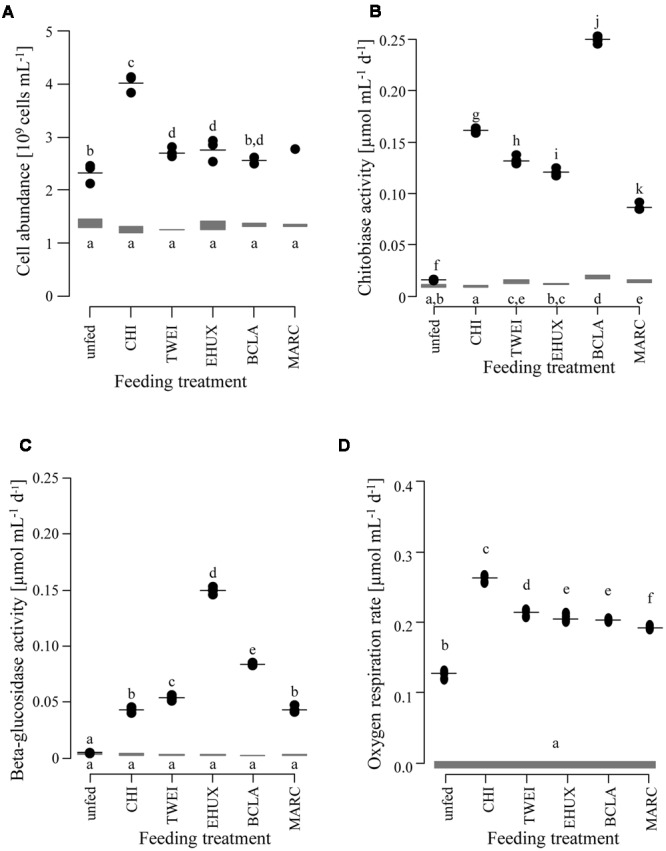
**Changes in cell abundance** (**A**; *n* = 3), extracellular enzyme activity (**B**: chitobiase, **C**: beta-glucosidase; *n* = 5), and oxygen respiration rates (**D**; *n* = 5) of the sediment community in the different treatments under atmospheric pressure conditions. Gray bars show the range at the beginning of the incubation. Black dots show measurements after 23 days of incubation, with the horizontal black line indicating the mean value per treatment. Lower case letters indicate groups of treatments that are significantly different from each other based on Tukey HSD at a significance threshold of *p* < 0.05. Day 0: starting conditions, unfed: control sediments after 23 days of incubation, carbon amendments: chitin (CHI), *Thalassiosira weissflogii* (TWEI), *Emiliania huxleyi* (EHUX), *Bacillaria* sp. (BCLA), *Melosira arctica* (MARC). Only one measurement of cell abundance was available for MARC-treated sediment.

Prokaryotic cell numbers in sediments retrieved from the central HAUSGARTEN station were on average 1.4±0.2 × 10^9^ cells (mL sediment)^-1^ (*n* = 3). After 23 days of incubation, cell numbers increased in all treatments, and in the unfed control, with highest cell numbers of about 4 × 10^9^ cells mL^-1^ in the CHI treatment (ANOVA, *F*_1,9_ = 152.7, *p* < 0.05) (**Figure 1A**; **Table 1**). Accordingly, bacteria in CHI treatments showed the highest estimated assimilation of carbon into biomass, i.e., 17% of the supplied carbon, while bacteria assimilated around 8% of the supplied carbon in MARC treatments, and ≤5% in the remaining algae treatments (**Table 1**). Based on the cell numbers, estimated duplication times ranged between 33 days for the control incubation and 12 days for the CHI-treated samples. The unfed control may serve as an approximation of community doubling times in Arctic deep-sea surface sediments, which can thus be estimated to about four weeks. The corresponding biomass calculations represent rough estimates of carbon conversion into bacterial biomass, as we did not quantitatively determine changes in cell volumes.

**Table 1:** Carbon utilization in the different treatments at atmospheric pressure.

	Treatment	Cell abundance [×10^9^ cells mL^-1^]	Cell biomass [μmol C mL^-1^]^a^	Stimulated net bacterial biomass yield [μmol C ml^-1^]^b^	C assimilation into biomass [% of added C]^c^	O_2_ uptake in 23 days [μmol O_2_ mL^-1^]	Net C respiration in 23 days [μmol C mL^-1^]^d^	Net C respired in 23 days [% of added C]^e^	Total C used in 23 days (respired + assimilated) [μmol mL^-1^]^f^	Total C used [% of added carbon]^g^	Bacterial growth efficiency [%]^h^
day0	unfed	1.4 ± 0.1	2.3 ± 0.1								
	CHI	1.3 ± 0.1	2.1 ± 0.1								
	TWEI	1.3 ± 0.0	2.1 ± 0.0								
	EHUX	1.4 ± 0.1	2.3 ± 0.1								
	BCLA	1.4 ± 0.0	2.3 ± 0.1								
	MARC	1.4 ± 0.0	2.3 ± 0.0								
day23	unfed	2.3 ± 0.2	3.9 ± 0.3	n/a	n/a	2.9 ± 0.1	n/a	n/a	n/a	n/a	n/a
	CHI	4.0 ± 0.1	6.7 ± 0.2	2.8 ± 0.4	17.0 ± 2.3	6.1 ± 0.1	3.2 ± 0.1	18.7 ± 0.5	6.0 ± 0.4	36.0 ± 2.3	47.2 ± 3.3
	TWEI	2.7 ± 0.1	4.5 ± 0.1	0.7 ± 0.4	3.9 ± 1.0	4.9 ± 0.1	2.0 ± 0.1	11.9 ± 0.5	2.7 ± 0.1	16.0 ± 0.8	23.8 ± 4.6
	EHUX	2.8 ± 0.2	4.6 ± 0.3	0.6 ± 0.2	4.4 ± 2.5	4.7 ± 0.1	1.8 ± 0.1	10.6 ± 0.6	2.5 ± 0.3	15.3 ± 2.0	27.0 ± 13.7
	BCLA	2.6 ± 0.1	4.3 ± 0.1	0.4 ± 0.2	2.4 ± 0.9	4.7 ± 0.0	1.8 ± 0.0	10.3 ± 0.1	2.1 ± 0.2	12.7 ± 0.9	18.6 ± 5.5
	MARC	2.8 ± n/a	4.6 ± n/a	1.1 ± n/a	6.5 ± n/a	4.4 ± 0.0	1.5 ± 0.0	8.7 ± 0.2	2.5 ± n/a	15.1 ± n/a	42.8 ± n/a

*All values are calculated as carbon content or oxygen uptake per mL wet sediment. The total carbon added for the different detritus treatments was equivalent to 16.6 μmol carbon mL^-1^ sediment. Day 0: starting conditions (*in situ* community), unfed: control sediments after 23 days of incubation, carbon amendments: chitin (CHI), *Thalassiosira weissflogii* (TWEI), *Emiliania huxleyi* (EHUX), *Bacillaria* sp. (BCLA), *Melosira arctica* (MARC); C: carbon; n/a: not applicable.*

*^a^Using a conversion factor of 1.67 fmol C per cell (Børsheim et al., 1990; Boetius and Lochte, 1996), representing an estimate of bacterial biomass; ^b^bacterial biomass yield in carbon-amended treatments minus bacterial biomass yield in unfed control; ^c^biomass yield divided by carbon added; ^d^respiration in carbon-amended treatments minus respiration in unfed control as derived from oxygen uptake, and assuming a conversion factor of 1; ^e^respired carbon divided by carbon added; ^f^sum of respired and assimilated carbon; ^g^total carbon used divided by total carbon added; ^h^biomass yield divided by total carbon used (assimilated + respired).*

### Enzymatic Activity

Chitobiase and beta-glucosidase enzymatic activities increased significantly in all carbon-amended samples, while enzymatic activities in the unfed control remained low (approximately 0.01 μmol mL^-1^ d^-1^), and were close to starting conditions (chitobiase: ANOVA, *F*_11,48_ = 5,811, *p* < 0.05; beta-glucosidase: ANOVA, *F*_11,48_= 5,297, *p* < 0.05; **Figures 1B,C**). Highest chitobiase activity was measured in the BCLA-fed sediments with 0.25 μmol mL^-1^ d^-1^, which was 16-fold higher than in the unfed control, followed by CHI amended samples with 0.16 μmol mL^-1^ d^-1^. The remaining algae-fed communities showed a moderate increase in chitobiase activity (0.10–0.13 μmol mL^-1^ d^-1^). All carbon treatments were significantly different from the unfed control and from each other (Tukey HSD, *p* < 0.05; **Figure 1B**). Highest beta-glucosidase activity was induced in the EHUX treatments with 0.15 μmol mL^-1^ d^-1^, which represents a 30-fold higher activity compared to the unfed control. Lowest beta-glucosidase activity was induced in the CHI and MARC treatments, turning over only 0.04 μmol mL^-1^ d^-1^. TWEI and BCLA addition resulted in moderate beta-glucosidase activities. All carbon treatments were significantly different from the unfed control and from each other (Tukey HSD, *p* < 0.05; **Figure 1C**), except for beta-glucosidase activity in the CHI and MARC treatments. Calculation of the total hydrolysis potential of both measured enzymes over the duration of the experiment showed that communities in BCLA and EHUX treatments had the highest potentials (52 and 38 μmol C mL^-1^ d^-1^, respectively), whereas MARC treatments had the lowest (17 μmol C mL^-1^ d^-1^).

### Oxygen Uptake

Over the 23 days of incubation, oxygen concentrations declined significantly in all treatments (ANOVA, *F*_6,28_ = 3,381, *p* < 0.05; **Figure 1D**; **Table 1**). Inferred oxygen uptake among the treated sediment slurries ranged between 6.1 μmol O_2_ mL^-1^ and 4.4 μmol O_2_ mL^-1^ sediment over 23 days for CHI and MARC treatments, respectively (**Table 1**). The unfed control showed lowest oxygen uptake of 2.9 μmol O_2_ mL^-1^ sediment in 23 days. Based on the calculated oxygen uptake, 19 and 9% of the added carbon have been respired at the end of the incubation period for CHI and MARC treatments, respectively (**Figure 1D**; **Table 1**). Oxygen uptake calculated for the addition of the planktonic algae and BCLA were equally moderate, with approximately 10% of the added carbon being respired (**Table 1**).

### Bacterial Diversity and Community Structure (16S rDNA and rRNA)

#### General Trends

**FIGURE 2:**
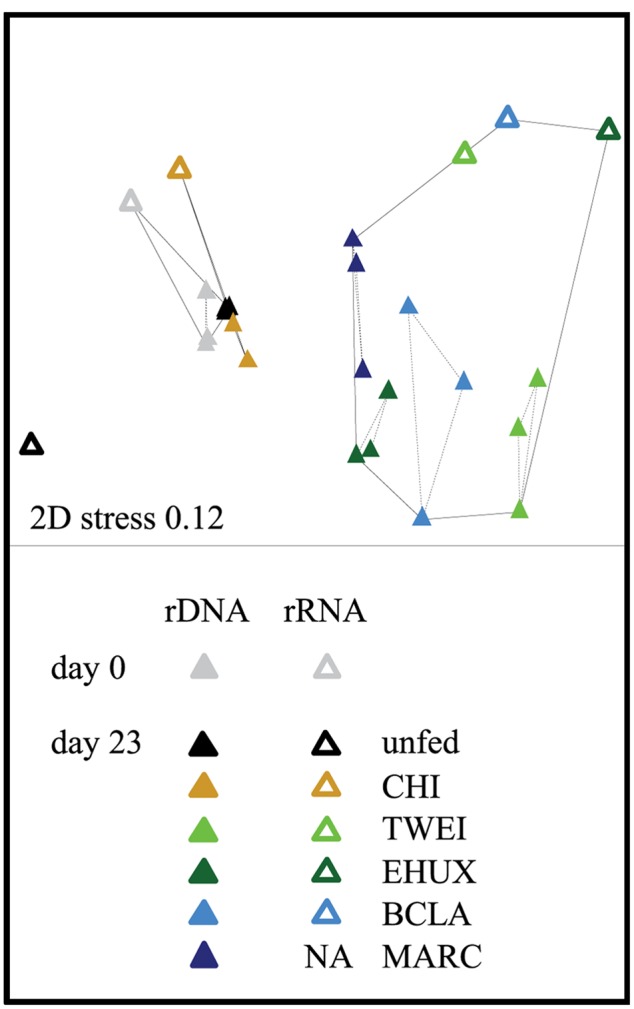
**Non-metric multidimensional scaling (NMDS) plot based on Bray–Curtis dissimilarity of the total (16S rDNA; *n* = 3) and active (16S rRNA; *n* = 1) bacterial community in the different sediment treatments under atmospheric pressure conditions.** Hulls displayed by solid lines are based on a dissimilarity threshold of 70%. Hulls displayed by dashed lines are based on a dissimilarity threshold of 32%, and shows clusters of biological replicates. Day 0: starting conditions, unfed: control sediments after 23 days of incubation, carbon amendments: chitin (CHI), *Thalassiosira weissflogii* (TWEI), *Emiliania huxleyi* (EHUX), *Bacillaria* sp. (BCLA), *Melosira arctica* (MARC). No rRNA data is available for MARC-treated sediment.

In total, 32,502–277,806 rDNA and 26,712–125,131 rRNA reads were generated per sample, corresponding to 1,731–12,549, and 3,012–16,384 swarmed, non-singleton OTUs, respectively. Changes in community structure were assessed separately for the 16S rDNA and rRNA sequence datasets, and samples from the two different approaches had an average intra-sample Bray–Curtis dissimilarity of 30% on family level, and 50% on OTU level. However, overall variations in community structure between treatments were significantly correlated between 16S rDNA and rRNA data on OTU and family level (**Figure 2**; Mantel test based on Bray-Curtis dissimilarity, *r* = 0.7, *p* < 0.05 for both taxonomic levels). Also, variations in community structure were highly correlated between experiments at atmospheric and at *in situ* pressure of 250 atm (Mantel test based on Bray–Curtis dissimilarity, *r* = 0.9, *p* < 0.05 on OTU and family level for rRNA and rDNA). Because data from biological replicates were available, we mainly focus on rDNA results under atmospheric pressure conditions here. Differences in the trends between rDNA and rRNA datasets are explicitly mentioned.

Bacterial communities were most diverse at the start of the incubation, with an observed bacterial richness of 5,777 OTUs and an inverse Simpson diversity index of 359 (**Table 2**). Diversity decreased significantly after 23 days in all treatments (OTU number: ANOVA, *F*_6,13_ = 136.8, *p* < 0.05; inverse Simpson index: ANOVA, *F*_6,13_ = 345.3, *p* < 0.05; **Table 2**), but not in control sediments. After 23 days of incubation, effective species richness was highest in the unfed samples with an inverse Simpson index of 193, followed by the CHI treatments with 56. Phytodetritus treatments resulted in the lowest alpha-diversity, with inverse Simpson indices ranging between 25 and 38.

**Table 2:** Alpha diversity indices of the bacterial community in the incubation experiment.

		nOTUs		invS	
	Treatment	Value	Tukey HSD	Value	Tukey HSD
**rDNA**
	day0	5,777 ± 56	a	359 ± 28	a
	unfed	5,113 ± 54	a	193 ± 22	b
	CHI	3,479 ± 359	b	56 ± 8	c
	TWEI	1,323 ± 95	c	26 ± 0	d
	EHUX	1,786 ± 44	d	25 ± 10	d
	BCLA	1,686 ± 286	d	35 ± 1	e
	MARC	1,922 ± 131	d	38 ± 2	e
**rRNA**
	day0	7,816	NA	1,230	NA
	unfed	2,767	NA	45	NA
	CHI	4,602	NA	67	NA
	TWEI	2,362	NA	32	NA
	EHUX	2,358	NA	34	NA
	BCLA	2,498	NA	34	NA
	MARC	NA	NA	NA	NA

*Richness and evenness were estimated based on OTU number (nOTU) and the inverse Simpson index (invS) of rDNA (*n* = 3) and rRNA (*n* = 1) datasets, and are given as mean ± standard deviation where applicable. Differences between treatments were assessed with ANOVA at a significance threshold of 0.05. Letters indicate significantly different groups based on pairwise Tukey HSD *post hoc* test. Day 0: starting conditions, unfed: control sediments after 23 days of incubation, carbon amendments: chitin (CHI), *Thalassiosira weissflogii* (TWEI), *Emiliania huxleyi* (EHUX), *Bacillaria* sp. (BCLA), *Melosira arctica* (MARC).*

**FIGURE 3:**
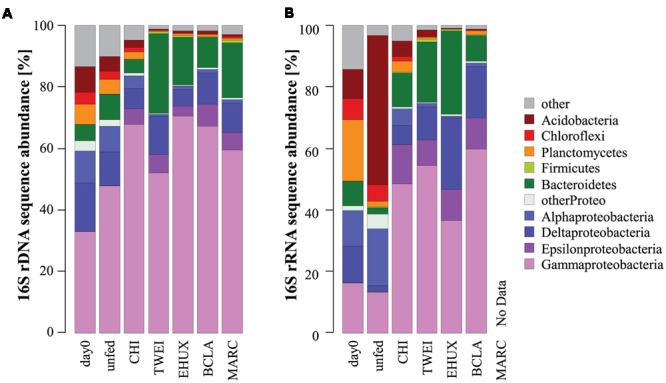
**Dominant phyla of the total** (**A**: 16S rDNA; *n* = 3) and active (**B**: 16S rRNA; *n* = 1) bacterial community in the different sediment treatments under atmospheric pressure conditions. For the total bacterial community, sequences from replicate samples were pooled for the calculation of relative sequence abundances. For *Proteobacteria*, class-level resolution is shown. Day 0: starting conditions, unfed: control sediments after 23 days of incubation, carbon amendments: chitin (CHI), *Thalassiosira weissflogii* (TWEI), *Emiliania huxleyi* (EHUX), *Bacillaria* sp. (BCLA), *Melosira arctica* (MARC).

The sediment community at the start of the incubation was dominated by *Proteobacteria*, comprising more than 60% of 16S rDNA sequences, and by members of the *Bacteroidetes, Planctomycetes, Chloroflexi*, and *Acidobacteria*, all of which showed relative sequence abundances between 5 and 10% (**Figure 3**). An NMDS plot based on Bray–Curtis dissimilarity revealed three distinct groups of samples at a 70% dissimilarity threshold (**Figure 2**). Bacterial community structure in the unfed control on day 23 was still very similar to the initial bacterial community. CHI treatments grouped separately, but were still similar to the unfed treatments. Phytodetritus treatments formed a third group, which was clearly separated from the unfed control and CHI treatments (**Figure 2**).

**FIGURE 4:**
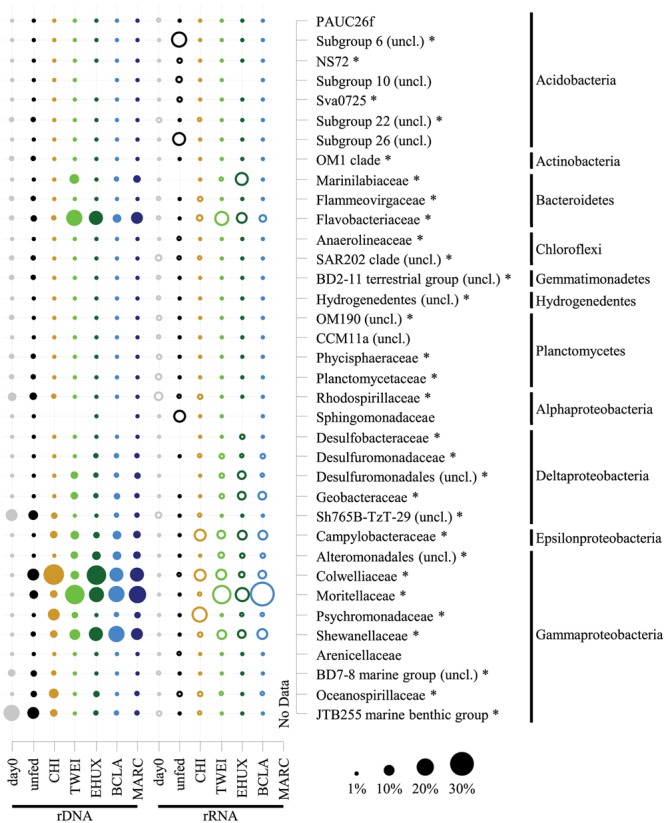
**Dot plot showing relative sequence abundances of dominant bacterial families, and their phylum affiliation, of the total (16S rDNA; *n* = 3) and active (16S rRNA; *n* = 1) bacterial community in the different sediment treatments under atmospheric pressure conditions.** For the total bacterial community, sequences from replicate samples were pooled for the calculation of relative sequence abundances. For families of the *Proteobacteria*, class-level resolution is shown. For taxa that were unclassified at the respective level of resolution, the next higher taxonomic rank is shown. Asterisks mark differentially abundant taxa between treatments based on 16S rDNA samples (ALDEx2 analysis). All groups that are not marked by an asterisk were only abundant in 16S rRNA data, for which no replicates are available and therefore no analysis of differential abundance could be performed. Day 0: starting conditions, unfed: control sediments after 23 days of incubation, carbon amendments: chitin (CHI), *Thalassiosira weissflogii* (TWEI), *Emiliania huxleyi* (EHUX), *Bacillaria* sp. (BCLA), *Melosira arctica* (MARC). No rRNA data is available for MARC-treated sediment.

Overall, 30 bacterial families of eight different phyla had a relative sequence abundance, i.e., sequence proportion >2% in at least one sample, and were identified as differentially abundant on rDNA level (**Figure 4**). About one third of these differentially abundant families (12 families), which were mainly affiliated with *Gamma*- and *Deltaproteobacteria*, and *Bacteroidetes*, increased significantly during incubations, whereas the other two thirds (18 families) decreased. The 12 families, which increased most strongly in relative sequence abundance in the different treatments, were identified as key responding, opportunistic bacterial families. These included four families responding to all treatments, including the unfed control: *Colwelliaceae, Moritellaceae, Psychromonadaceae*, and *Shewanellaceae*. In addition, four families responded to all carbon amendments: *Desulfuromonadaceae*, unclassified *Desulfuromonadales* and *Alteromonadales* families, as well as *Campylobacteraceae*. Another three families showed a strong positive response in relative sequence abundance especially to the supply of phytodetritus: *Flavobacteriaceae, Geobacteraceae*, and *Marinilabiaceae*. The *Oceanospirillaceae* increased significantly in the unfed control treatment, on average sixfold stronger than in any other treatment except for the CHI sample. Furthermore, minor changes in the unfed control treatment included an increase in relative sequence abundance of four gammaproteobacterial families, and a slight decrease of two families belonging to the *Chloroflexi* and *Acidobacteria*. Otherwise, no changes in the majority of the most abundant families were detected (**Figure 4**). In total, the 12 opportunistic families increased from 3% relative sequence abundance at the beginning of the incubation to on average 89% in carbon treatments after 23 days of incubation (Supplementary Tables S4A,B). The only case of pronounced differences between rDNA and rRNA results was the unfed control after 23 days. On the level of active transcription (rRNA), diverse families belonging to the phylum *Acidobacteria*, i.e., subgroups 6, 10, 26 (unclassified), NS72, and SVA0725, as well as the alphaproteobacterial family *Sphingomonadaceae* increased strongly in relative sequence abundance. This was not the case on rDNA level, which still closely resembled the starting community composition.

#### Chitin Treatments

Overall, a different set of families responded to the CHI treatment compared to the phytodetritus treatments. CHI treatment led to a significant increase in nine out of the 30 differentially abundant families, whereas three decreased compared to the initial sediment community. In contrast to phytodetritus treatments, *Colwelliaceae, Psychromonadaceae*, and *Oceanospirillaceae* were most strongly affected by CHI treatments, increasing from < 1% of 16S rDNA relative sequence abundance to 25, 12, and 8%, respectively (**Figure 4**; Supplementary Table S4A). Overall, most bacterial families with higher relative sequence abundances in the unfed controls, e.g., *Oceanospirillaceae*, or in phytodetritus treatments, e.g., *Campylobacteraceae* and *Desulfuromonadaceae*, also increased in relative abundance in CHI treatments (**Figure 4**).

#### Phytodetritus Treatments

All phytodetritus treatments led to stronger changes in community structure than observed for unfed controls and CHI treatments after 23 days of incubation (**Figure 2**). On average, eleven families increased and 15 decreased in relative sequence abundance in phytodetritus treatments, and they were very similar in their phylogenetic affiliation across all treatments (**Figure 4**; Supplementary Table S4A). The families most strongly increasing in relative sequence abundance for the different algal treatments were *Flavobacteriaceae, Moritellaceae*, and *Desulfobacteraceae* for TWEI, *Marinilabiaceae*, unclassified *Desulfuromonadales* families, and *Geobacteraceae* for TWEI as well as EHUX, and an unclassified group of *Alteromonadales* for EHUX. *Desulfuromonadaceae*, Shewanellaceae, and *Campylobacteraceae* responded strongest to BCLA. All of the abundant families classified as opportunistic in algal treatments (EHUX, TWEI, BCLA) also increased significantly in MARC treatments.

#### Response of Bacterial Groups at High Taxonomic Resolution

The analysis of differentially abundant bacterial groups at a higher taxonomic resolution, i.e., at OTU level, revealed intra-genus differences in their response to the different treatments in four out of the 30 differentially abundant families. Within the genera *Arcobacter* (*Campylobacteraceae*), *Colwellia* (*Colwelliaceae*), *Moritella* (*Moritellaceae*) and *Psychromonas* (*Psychromonadaceae*), several OTUs displayed opposing trends among the treatments, in addition to the overall pattern observed at lower taxonomic resolution (SI text).

## Discussion

Environmental changes in the Arctic Ocean lead to changes in the quality and quantity of organic matter exported from surface waters to the deep sea (McMahon et al., 2006; Bauerfeind et al., 2009; Boetius et al., 2013), but little is known about the effects on benthic community structure and function. In this study, we aimed at evaluating the response of benthic bacterial communities of the Arctic deep-sea floor to different organic matter sources in short-term, *ex situ* experiments. Using an experimental setup allowing for approximately one community duplication (**Table 1**), we were able to observe changes in community functions and, for the first time, identified bacterial groups that responded within days to the addition of different naturally occurring organic matter sources, i.e., chitin and different species of algae.

Responses in growth, extracellular enzymatic activities, and oxygen uptake were observed for all organic matter treatments compared to unfed control incubations after 23 days. This showed that bacterial communities actively responded to the input of organic matter by initiating degradation of the organic matter and producing biomass. This observation is supported by previous studies from various deep-sea environments (Deming, 1985; Turley and Lochte, 1990; Boetius and Lochte, 1994, 1996; Witte et al., 2003a). Although carbon uptake appeared to be slightly lower for incubations under *in situ* pressure (see also: Jannasch, 1979; Jannasch and Wirsen, 1982), oxygen consumption was higher, resulting in hypoxic conditions after 23 days. Despite this discrepancy, similar results were obtained for incubations at atmospheric and *in situ* pressure (**Figures 1**–**4**; Supplementary Figures S2–S6), of which the latter need to be interpreted with caution, as we cannot clearly distinguish between the effects of pressure and oxygen depletion in the high-pressure treatments.

In addition to the response in bulk growth and activity measurements, we observed clear shifts in diversity (**Table 2**) and community structure (**Figure 2**) as a result of organic matter additions. The responding taxa were generally consistent between rDNA and rRNA, indicating that shifts in bacterial community structure quickly translated from a higher activity (rRNA) to cell replication (rDNA). Furthermore, changes in community structure were largely consistent between incubations at 1 and 250 atm, indicating that most of the opportunistic bacterial groups appear to cope well with de-/re-compression for the investigated water depth (2,500 m). Accordingly, pressure effects have repeatedly been hypothesized to be negligible below approximately 200–300 atm (Yayanos, 1986; Follonier et al., 2012; Picard and Daniel, 2013). Obligate piezophilic bacteria mainly derive from below 6,000 m water depth (Yayanos, 1986), and most bacterial groups from shallower water depths are described to be rather piezotolerant (Picard and Daniel, 2013).

### Changes in the Untreated Control Community

For both, atmospheric and *in situ* pressure incubations, an increase in bacterial biomass in control treatments indicated that the presence of sedimentary organic carbon provided sufficient energy for an increase of the bacterial standing stock. This may be partly due to the onset of a settling phytoplankton bloom in the area during the time of sampling (Soltwedel, 2015), but also to the presence of refractory organic material. The initial (*in situ*) sediment bacterial community (**Figure 3**) was very similar to previous reports from this site (Jacob et al., 2013), and from other deep-sea surface sediments globally (Zinger et al., 2011; Bienhold et al., 2016; Learman et al., 2016). OTUs affiliating with the gammaproteobacterial families *Oceanospirillaceae* and *Colwelliaceae* showed a strong increase in relative sequence abundance in the unfed control treatments after 23 days of incubation, but also in CHI treatments. These groups are known to include polymer degraders (Methé et al., 2005; Weiner et al., 2008). Additionally, the genera *Colwellia* and *Marinomonas* sp. (*Oceanospirillaceae*) have been reported to produce polyhydroxyalkanoate compounds, which serve as intracellular carbon and energy reserves (Methé et al., 2005; Cai et al., 2011). This may be a beneficial strategy for organisms living in organic-poor sediments of the Arctic deep sea.

We also observed a conspicuous increase in relative rRNA sequence abundance of deep-branching acidobacterial groups and taxa belonging to the *Sphingomonadaceae* in the unfed controls after 23 days of incubation. We speculate that the increased relative rRNA sequence abundance may represent a strategy, where in lack of nutrients, energy is invested in maintaining the metabolism rather than increasing biomass (Morita, 1982; Watson et al., 1998 and references therein). However, this pattern was not reproducible in incubations at 250 atm, and it remains unknown what caused this discrepancy. Due to the lack of fresh organic material in control incubations, bacteria adapted to more oligotrophic conditions and the breakdown of refractory, aged organic material may become more important. Indeed, *Sphingomonadaceae* are described to play important roles in oligotrophic environments, and in the degradation of recalcitrant polyaromatic compounds (Ohta et al., 2004; García-Romero et al., 2016). In spite of their frequently observed oligotrophic character, members of this family are widespread in nature, occurring in soils, freshwater, and marine habitats (Cavicchioli et al., 1999; Yabuuchi and Kosako, 2005; Vaz-Moreira et al., 2011). *Acidobacteria* have also been reported to cope well with low organic matter availability (Fierer et al., 2007; Bienhold et al., 2012). These groups may thus be representatives of a background community, surviving in the oligotrophic conditions that prevail at the deep Arctic seafloor for most of the year.

### Effect of Detritus-Type on Bacterial Community Structure and Function

Chitin addition caused strong functional changes in the sediment microbial community, i.e., the largest increase in net biomass (highest bacterial growth efficiency), highest oxygen consumption rates, and a strong increase in chitobiase activity (**Figure 1**; **Table 1**). Despite these effects, and even though overall diversity decreased significantly (**Table 2**), community composition remained very similar to its initial structure and to control incubations (**Figure 2**; Supplementary Figure S4). The sediment community thus seemed to be well adapted to the degradation of this type of polymeric organic matter. Since chitin is the most abundant biopolymer in the ocean (Gooday, 1990), and bacteria are the main decomposers of this material in the deep-sea benthos (Christiansen and Boetius, 2000; Kanzog et al., 2009), benthic communities may constantly be exposed to relatively high levels of chitin, and our results may therefore reflect an inherent adaptation to the degradation of this carbon source. The decrease in community diversity probably reflects the loss of taxa that were overgrown by chitin degraders.

In contrast to CHI treatments, the addition of algae caused a much more moderate response in bulk community function, including lower estimated bacterial growth efficiencies. This is in line with reports by Mayor et al. (2012), who showed that benthic bacteria displayed lower growth efficiencies when respiring diatoms compared to fecal pellet material, and confirms that resource quality may influence carbon uptake and retention potential in natural settings.

There are several potential explanations for the lower bacterial growth efficiencies in algae treatments. Some of the tested algae may contain compounds that suppress growth of certain bacterial groups (Guedes et al., 2011; Qin et al., 2013). As one example, the production of dimethylsulphopropionate (DMSP)-related substances by EHUX has been suggested to act as protection against grazers (Hansen et al., 1996; Fileman et al., 2002), and may also inhibit bacterial attachment (Saha et al., 2012). Viral lysis might act as another structuring factor (Proctor and Fuhrman, 1990; Fuhrman and Noble, 1995), as viruses are also considered to be favored by high energy input and biological productivity (Cochlan et al., 1993; Bratbak et al., 1994). Furthermore, meiofauna, e.g., nematodes, may be direct or indirect beneficiaries of the fresh phytodetritus, by either feeding on the algae or on the bacteria (Guilini et al., 2010; Ingels et al., 2010). Numerous studies have shown that fauna may be rapid consumers of fresh phytodetritus at the seafloor (e.g., Blair et al., 1996; Moodley et al., 2002, 2005, Witte et al., 2003a), in particular in the Arctic, following the deposition of ice algae or phytoplankton (McMahon et al., 2006; Morata et al., 2011, 2015). To further resolve the substrate degradation cascade, labeled substrates could be used in future experiments (Witte et al., 2003a,b; Guilini et al., 2010).

Compared to CHI treatments, we observed a much stronger decrease in bacterial diversity as well as shifts in community structure in all phytodetritus treatments, indicating the response of a few adapted groups outcompeting others. This suggests that changes in community composition needed to precede changes in community function (Schindler, 1987; Niederlehner and Cairns, 1994). This may be due to the fact that the input of relatively fresh algae used in this study is less common than the ubiquitously available chitin. Also, while chitin is solely composed of *N*-acetyl-glucosamine subunits, and only a small set of enzymes is necessary to degrade it, the degradation of algae is more complex, involving a cascade of multiple enzymes and bacterial groups (Xing et al., 2015; Teeling et al., 2016).

Changes in community structure and function were very similar between the phytoplankton (TWEI, EHUX) and sea-ice algae (BCLA, MARC) treatments, despite presumable differences in their nutritional value (McMahon et al., 2006; Sun et al., 2007). However, the input dynamics of these organic matter sources in the natural system would differ profoundly. Specifically, a slow rain of phytoplankton cells that are already being degraded on their way through the water column represent a stark contrast to a highly concentrated and localized input of large ice algae aggregates sinking rapidly to the deep seafloor. These scenarios would likely result in differential impacts on community structure, functioning, and carbon turnover.

### Taxon-Specific Responses to Carbon Amendments

Previous studies have already identified links between organic matter quantity and bacterial community structure in marine sediments (Franco et al., 2007; Bienhold et al., 2012; Learman et al., 2016). Here, we specifically addressed the response of bacterial communities to different types of organic matter when provided in a single, large pulse. Overall, we identified 12 bacterial families on both rRNA and rDNA level that increased in relative sequence abundance in the organic matter treatments, and that were consistent between incubations under atmospheric and *in situ* pressure conditions. These opportunistic bacterial groups may represent the specific community fraction that contributed to the increase in enzyme activity, biomass, and oxygen respiration, and that might thus be of key importance for initial polymer degradation at the deep-sea floor. The gammaproteobacterial families *Colwelliaceae, Psychromonadaceae*, and *Oceanospirillaceae* were found in highest abundances in CHI treated sediments. *Gammaproteobacteria* are a globally ubiquitous group in marine environments that have been described as versatile opportunists and copiotrophs (Glöckner et al., 1999; Bienhold et al., 2012). Indeed, representatives of the family *Oceanospirillaceae*, e.g., the genera *Marinomonas* and *Neptunomonas*, have been described to degrade chitin (Nogi et al., 2004; González and Whitman, 2006; Jung, 2006). Also representatives of the families *Colwelliaceae* and *Psychromonadaceae* appear to have a broad substrate spectrum including complex organic compounds (Methé et al., 2005; Auman et al., 2006). Several members of the *Colwelliaceae* have been reported to hydrolyze chitin (Huston et al., 2000, 2004; Ivanova et al., 2004). We therefore identify these bacterial groups as potential opportunists, especially in scenarios of high chitin input, such as mass sedimentation events of crustaceans (Sokolova, 1994; Christiansen and Boetius, 2000).

For *Psychromonadaceae*, only some species are described as being able to degrade chitin (e.g., strain *Psychromonas ingrahamii* 37; Riley et al., 2008), whereas other species, e.g., *Psychromonas arctica*, appear to lack this ability (Groudieva et al., 2003). This supports our findings of a strong increase in relative sequence abundance for diverse OTUs affiliated with *Psychromonas* in CHI amended slurries on the one hand, and a decrease of other *Psychromonas*-associated OTUs on the other hand, which instead showed a stronger response in algae treatments (SI text). This deviation is consistent with previous reports for this genus in a similar environment (Bienhold et al., 2012), and demonstrates the usefulness of community information at a high taxonomic resolution. In the future, genomic and physiological studies may reveal in more detail the different metabolic niches that these taxa occupy.

Several other families were identified with most pronounced shifts in the algae treatments. *Flavobacteria* from Arctic sediments were recently described to exhibit a strong positive correlation with energy availability (Bienhold et al., 2012), consistent with the association of copiotrophy to the phylum *Bacteroidetes* (Fierer et al., 2007). Also, *Flavobacteria* and *Alteromonadales*, in particular *Pseudoalteromonadales*, have shown strong responses to phytoplankton blooms (Teeling et al., 2016; Xing et al., 2015). Representatives of the order *Pseudoalteromonadales*, e.g., *Pseudoalteromonas haloplanktis*, which shares closest sequence identity to the responsive clade in our study, can produce anti-biofilm molecules (Parrilli et al., 2016). It may be able to degrade extracellular polymeric substances produced by phytoplankton and ice algae like MARC by using its diverse set of enzymes, including peptidases, amylase, and alpha-glucosidase (Médigue et al., 2005). In the future, extended monitoring periods after the addition of organic matter, combined with metagenomic and metatranscriptomic approaches, may yield further insights into succession patterns, species competition, and niche adaptation, e.g., as observed for bacterial communities associated with phytoplankton blooms in the water column (Teeling et al., 2012).

Most of the opportunistic bacterial groups identified in this study contain psychrophilic and psychrotolerant cultured representatives (Médigue et al., 2005; Methé et al., 2005; Auman et al., 2006; Riley et al., 2008; Lee et al., 2012) with mostly heterotrophic lifestyles (e.g., Médigue et al., 2005; Methé et al., 2005), the potential for motility (e.g., Médigue et al., 2005; Auman et al., 2006; Lee et al., 2012), and biofilm formation (e.g., Médigue et al., 2005; Methé et al., 2005; Riley et al., 2008; Baek et al., 2015). The latter two features may aid the association with, and directed degradation of particulate organic matter in the deep sea (O‘Toole et al., 2000; Golyshin et al., 2002), but the relevance of such mechanisms remains speculative and will require further investigations. Most of the groups are not specific or exclusive for the sediment environment, but cover a wide range of natural habitats, and appear to have a rather cosmopolitan character (e.g., Médigue et al., 2005; Methé et al., 2005; Zhao et al., 2005; Auman et al., 2006; Xing et al., 2015). Nevertheless, many of the closely related cultured representatives were isolated from polar marine environments, and are described to produce cold-adapted enzymes, capable of degrading high-molecular-weight organic compounds, e.g., *Alteromonadales* (Médigue et al., 2005), *Colwelliaceae* (Methé et al., 2005), *Shewanellaceae* (Zhao et al., 2010), *Psychromonadaceae* (Riley et al., 2008), *Moritellaceae* (Lee et al., 2012; Malecki et al., 2013), *Flavobacteriaceae* (Pati et al., 2011; Baek et al., 2015; Xing et al., 2015), and *Oceanospirillaceae* (Weiner et al., 2008). Future research may therefore identify explicit Arctic and/or sediment ecotypes within these groups, which need to be further metabolically characterized using genomic and physiological methods, in order to assess their role in the turnover of organic matter in Arctic marine sediments.

## Conclusion

The observed differences in community structure, carbon uptake, and remineralization between the treatments suggest that changes in the type of organic matter exported to the seafloor will have consequences for bacterial diversity and carbon turnover. The families *Colwelliaceae, Psychromonadaceae*, and *Oceanospirillaceae* may represent indicator groups for CHI input, and *Flavobacteriaceae, Pseudoalteromonadaceae*, and *Marinilabiaceae* for phytodetritus input in this Arctic region. However, quantitative methods targeting specific groups of interest, such as Catalyzed Reporter Deposition-Fluorescence *In Situ* Hybridization (CARD-FISH) and quantitative PCR, are needed to confirm the changes observed in the compositional sequencing datasets, and to further link taxonomic groups (at high taxonomic resolution) to specific community functions. Furthermore, the metabolic potential of these key responding groups remains to be investigated to better understand their role in ecosystem functioning. Future studies should combine *ex situ* and *in situ* observations at extended time periods. Experimental approaches will be helpful in order to monitor changes and succession patterns in community structure and function after disturbances. Additionally, long-term environmental observations are needed to better assess the effects of shifts in organic matter quality and quantity on ecosystem functioning in the context of seasonal variations and spanning several years.

## Author Contributions

KH and CB designed experiments. KH performed the experiments. KH, CH, and CB analyzed data, and VS-C assisted in data interpretation. MH performed oxygen sensor data analysis and modeling. KH, VS-C, and CB wrote the manuscript with support and input from all co-authors. All authors critically revised the article and gave their approval of the submitted version.

## Conflict of Interest Statement

The authors declare that the research was conducted in the absence of any commercial or financial relationships that could be construed as a potential conflict of interest.

## Footnotes

1 https://doi.org/10.1594/PANGAEA.867475

## Abbreviations

16S rDNA: 16S rRNA gene
BCLA: *Bacillaria* sp.
CHI: chitin
EEA: extracellular enzymatic activity
EHUX: *Emiliania huxleyi*
LTER: Long-Term Ecological Research Observatory
MARC: *Melosira arctica*
TWEI: *Thalassiosira weissflogii*
