# Supplementary material for: Response of Bacterial Communities to Different Detritus Compositions in Arctic Deep-Sea Sediments
Source: Front Microbiol. 2017 Feb 24;8:266. doi: 10.3389/fmicb.2017.00266 (PMC5323390; doi:10.3389/fmicb.2017.00266)
Supplement: Supplementary file 1 [file Data_Sheet_1.PDF]

Supplementary Material

Response of bacterial communities to different detritus compositions in Arctic deep-sea sediments

Katy Hoffmann[1,2] *, Christiane Hassenrück[1], Verena Salman-Carvalho[1,2], Moritz Holtappels[3], Christina Bienhold[1,2]

*** Correspondence: Katy Hoffmann**: khoffman@mpi-bremen.de

1 Supplementary Data

O_2 respiration experiments in permeable plastic bags

Plastic bags were filled with sediment slurries to a final volume (*V*) of 50 mL. The initial O_2 concentration (C_0) in the bags was 300 µmol L^{-1}. The bags were stored in a container filled with sterile filtered, air saturated seawater (C_0 = 300 µmol L^{-1}). The plastic material was O_2 permeable with a thickness (*x*) of 0.12 mm. From previous tests it was known that the O_2 diffusion coefficient (*D*) is 2.65 x 10^{-12} m² s^{-1}. The change of O_2 concentration over time in the bags was a function of the microbial respiration rate (*R*), which we assumed to be constant (zero order kinetics), and the O_2 flux (*F*) through the plastic foil of an area (*A*) of 81 cm². The flux, in turn, was a function of the different O_2 concentrations inside ($C_{(t)}$) and outside (C_0) the bags. This can be expressed by the equation:

$$\frac{dC}{dt} = R + \frac{(C_0 - C(t))}{x} D \, \frac{A}{V} \qquad (1)$$

Equation (1) can be solved for C

$$C(t) = \frac{R}{K}(1 - e^{-Kt}) + C_0 \qquad (2)$$

with the time constant *K* given by $$K = \frac{D\,A}{x\,V} \qquad (3)$$

In the bags, the O_2 concentrations decrease exponentially and approach a steady state (Supplementary Figure S1). After a long incubation time (i.e. $t >> K$) the O_2 respiration rate will be balanced by the inward flux of O_2 and the steady state solution is

$$C(t) = \frac{R}{K} + C_0 \qquad (4)$$

For the given volume (50 mL), area (81 cm²), diffusion coefficient (2.65 x 10^{-12} m² s^{-1}), and foil thickness (0.12 mm) the time constant *K* is 0.3 d^{-1}. This means that 63% of the total decrease of O_2 takes place within $1/K$ = 3.2 days. Consequently, the O_2 concentration is in steady state after 23 days

of incubation and the respiration rate can be calculated from the O_2 concentration at day 23 by rearranging equation (4)

$$R = (C_{23d} - C_0)K \qquad (5)$$

Response of bacterial groups at high taxonomic resolution

When analyzing differentially abundant bacterial groups at a higher taxonomic resolution, i.e. at OTU level, we identified intra-genus differences in their response to different treatments in four out of the 30 differentially abundant families. The family *Campylobacteraceae* consisted of one genus, *Arcobacter*, which strongly increased in all carbon-amended treatments. At a higher resolution, three different OTUs were assigned within this genus, of which two (OTU4 and 130) responded strongest to phytodetritus treatments, but OTU18 showed a 38% stronger relative sequence increase in CHI treatments compared to phytodetritus treatments (Supplementary Table S4). Similar observations were made for the gammaproteobacterial genera *Colwellia*, *Moritella*, and *Psychromonas*. Eight out of ten OTUs affiliating with the genus *Colwellia* responded to all treatments including the control. The *Colwellia* OTU22, however, showed a much stronger relative sequence response in the unfed control and EHUX treatment, i.e. a 57% stronger increase compared to all other treatments. *Colwellia* OTU12 increased by 71% in relative sequence abundance in the CHI and EHUX treatments, compared to other treatments. The genus *Moritella* included nine OTUs, of which seven showed a similar increase in relative sequence abundance in all treatments including the control, while OTUs 13 and 30 showed a 6 to 25% stronger increase in relative sequence abundance in TWEI and MARC treatments compared to all other treatments. The family *Psychromonadaceae* responded strongly to CHI treatments and was mainly represented by the genus *Psychromonas*, compared to the initial sediment community. While four out of five OTUs within this genus responded most strongly to CHI treatments in relative abundance, OTU55 responded much stronger to TWEI and MARC treatments, i.e. with a 99.9% higher increase in relative sequence abundance. A full list of differentially abundant OTUs is included in Supplementary Table S4.

2 Supplementary Tables and Figures

2.1 Supplementary Tables

Supplementary Table S1 Test of the oxygen permeability of the bag material (PE) in sea water. PE-bags were filled with a highly oxygen-reduced (N_2-purged) sterile slurry, and oxygen diffusion into the bags from the surrounding water in the incubators was monitored using optodes. Subsequently, equation (1) was used to back calculate the diffusion coefficient.

Measuring points [h]	$c(O_2)$ in bags [µmol L^{-1}]			Average [µmol L^{-1}]	O_2 difference [µmol L^{-1}]
	Replicate 1	Replicate 2	Replicate 3		
0 (start)	21	23	23	22.3	
1	32	37	33	34.0	11.7
2	43	44	43	43.2	9.2
3	47	51	45	47.7	4.4
5	61	60	63	61.3	6.8
7	71	73	74	72.4	5.5
10	85	90	87	87.6	5.1
12	95	99	100	98.0	5.2

Supplementary Table S2 Extracellular enzymes and their target substrates used in this study. Enzyme classifications and names correspond to the IUBMB enzyme nomenclature. The natural substrates represent examples of a variety of compounds hydrolysed by these enzymes.

Enzyme	Natural substrate (selection)	Artificial assay substrate	Final concentration of artificial substrate [µmol L^{-1}]
**Beta-glucosidase (E.C. 3.2.1.21)**	cellulose	4-methylumbelliferyl ß-D-glucopyranoside (MUF-ß)	100
**N-acetyl-ß-glucosaminidase (E.C. 3.2.1.30)**	chitin	4-methylumbelliferyl-N-acetyl-ß-D glucosaminide (MUF-NAc)	100

Supplementary Table S3 Alpha diversity indices of the bacterial community in the incubation experiment at 250 atm *in situ* pressure conditions. Sediment treatments: untreated sediment at the beginning of the incubation (day0), unfed control sediment after 23 days of incubation (unfed), sediments amended with chitin (CHI), *Thalassiosira weissflogii* (TWEI), *Emiliania huxleyi* (EHUX), *Bacillaria* sp. (BCLA), and *Melosira arctica* (MARC). Richness and evenness were estimated based on OTU number (nOTU) and the inverse Simpson index (invS) of rDNA (n= 3) and rRNA (n = 1) datasets, and are given as mean ± standard deviation where applicable. Differences between treatments were assessed with ANOVA at a significance threshold of 0.05. Letters indicate significantly different groups based on pairwise TukeyHSD post-hoc test.

		**nOTUs**		**invS**	
	treatment	value	TukeyHSD	value	TukeyHSD
**rDNA**					
	day0	5,777 ± 56	a	359 ± 28	a
	unfed	5,438 ± 139	a	259 ± 73	a
	CHI	2,842 ± 176	b	19 ± 8	b
	TWEI	1,092 ± 76	c	13 ± 0	b
	EHUX	1,693 ± 56	d	27 ± 10	b
	BCLA	1,494± 22	d	18 ± 1	b
	MARC	2,210 ± 157	e	27 ± 2	b
**rRNA**					
	day0	7,816	NA	1,230	NA
	unfed	7,352	NA	626	NA
	CHI	4,536	NA	30	NA
	TWEI	1,254	NA	16	NA
	EHUX	1,831	NA	25	NA
	BCLA	1,823	NA	18	NA
	MARC	2,962	NA	33	NA

Supplementary Table S4 can be found in the additional Excel File

Supplementary Table S4 Bacterial taxa potentially affected by treatments based on relative OTU abundances at (A) 1 atm and (B) 250 atm. For the rDNA dataset (n = 3) OTUs are shown, which were detected as differentially abundant between treatments at an adjusted parametric, and an unadjusted non-parametric significance threshold of 0.05, and further exhibited at least a doubling or a decrease by half, as well as an effect size larger than 4 between the start of the incubation and any of the sediment treatments at day 23. Additionally, OTUs from the rRNA (n = 1) dataset are shown, which constituted more than 1% sequence abundance in at least one sample, and exhibited at least a doubling or a decrease by half between the start of the incubation and any of the sediment treatments at day 23. Sediment treatments: untreated sediment at the beginning of the incubation (day0), unfed control sediment after 23 days of incubation (unfed), sediments amended with chitin (CHI), *Thalassiosira weissflogii* (TWEI), *Emiliania huxleyi* (EHUX), *Bacillaria* sp. (BCLA) and *Melosira arctica* (MARC).

2.2 Supplementary Figures

(A)

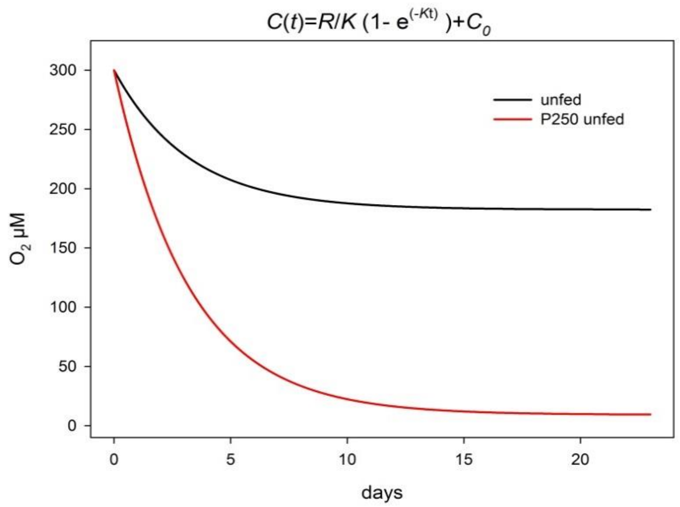

(B)

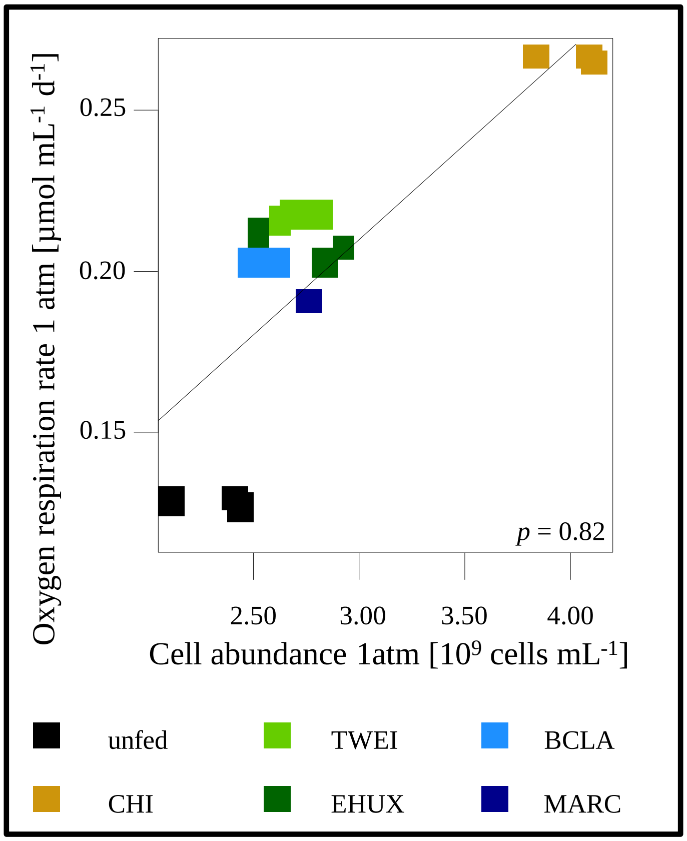

Supplementary Figure S1

(A) Modeled O_2 concentration [µM] in the incubated bags as a function of time (in days). Unfed control sediment after 23 days of incubation under 1 atm (unfed) and 250 atm (P250 unfed). (B) Correlation between cell abundance and oxygen respiration rate of the sediment community after 23 days of incubation for the different incubation treatments under atmospheric pressure conditions supporting the oxygen model. Absolute values (solid squares) are shown per replicate of each treatment (n = 3). The black line indicates a 1:1 ratio between each treatment for oxygen and cell abundance measurements; p = correlation of Pearson product-moment correlation coefficient. Sediment treatments: unfed control sediment after 23 days of incubation (unfed), sediments amended with chitin (CHI), *Thalassiosira weissflogii* (TWEI), *Emiliania huxleyi* (EHUX), *Bacillaria* sp. (BCLA) and *Melosira arctica* (MARC). Only one measurement of cell abundance was available for MARC-treated sediment.

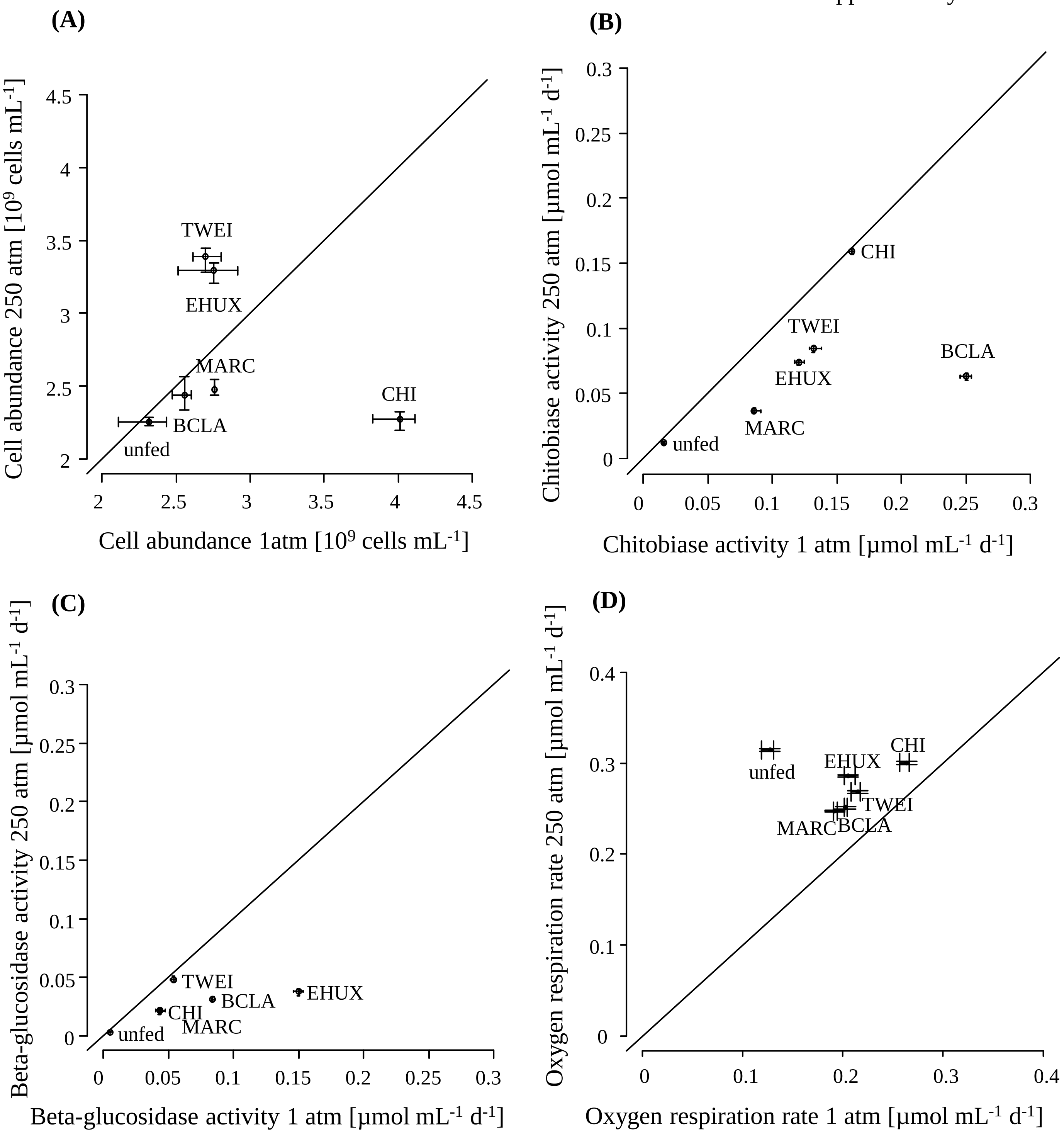

Supplementary Figure S2 Correlation of cell abundance (A; n = 3), extracellular enzymatic activity (B: chitobiase, C: beta-glucosidase; n = 5), and oxygen respiration rates (D; n = 5) of the sediment community after 23 days of incubation under atmospheric and 250 atm *in situ* pressure conditions. Mean values (solid circles) are shown with whiskers indicating the range per treatment. The black line indicates identical values under both pressure conditions. Sediment treatments: unfed control sediment after 23 days of incubation (unfed), sediments amended with chitin (CHI), *Thalassiosira weissflogii* (TWEI), *Emiliania huxleyi* (EHUX), *Bacillaria* sp. (BCLA) and *Melosira arctica* (MARC). Only one measurement of cell abundance was available for MARC-treated sediment. Only one measurement of cell abundance was available for MARC-treated sediment.

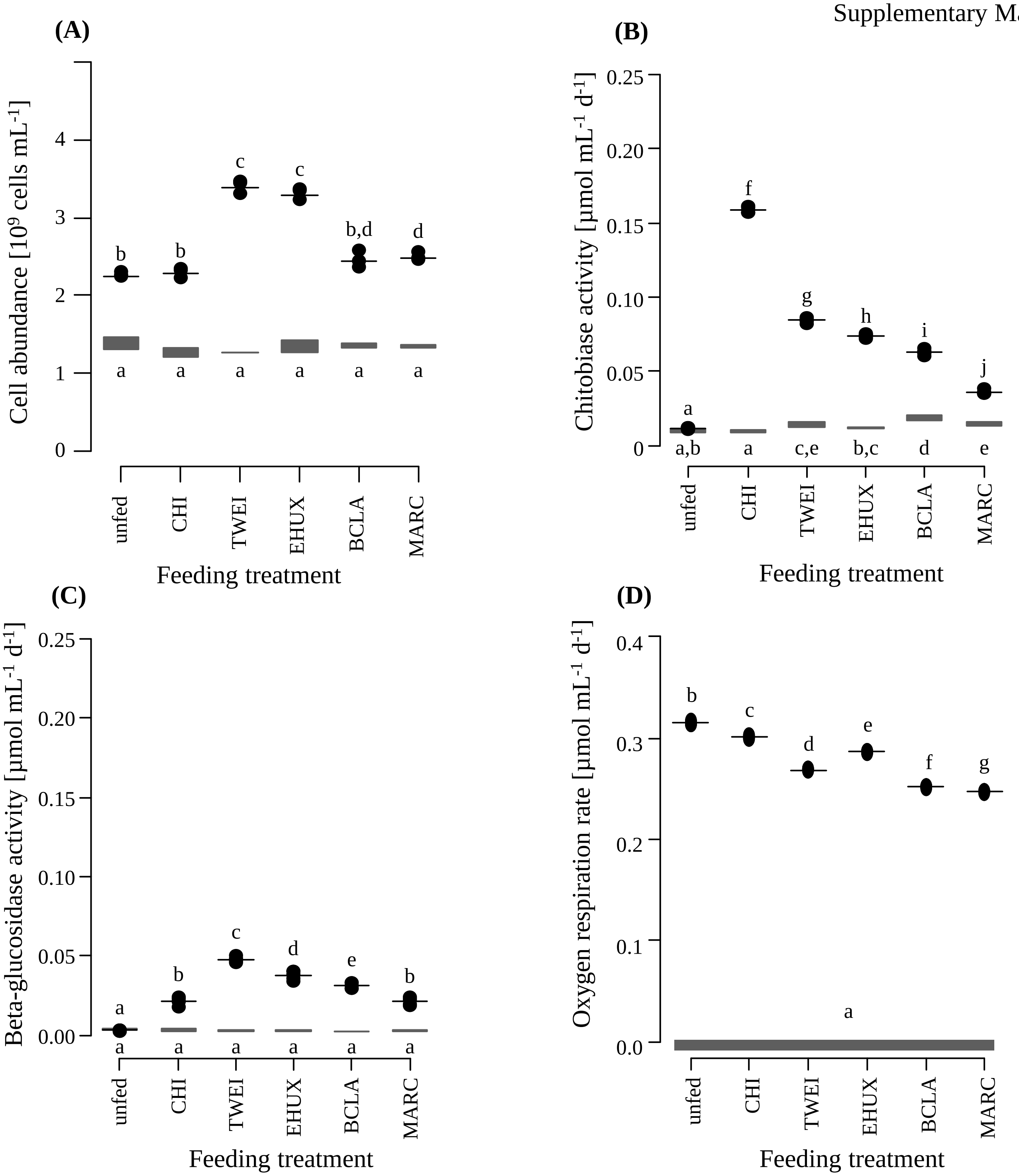

Supplementary Figure S3 Changes in cell abundance (A; n = 3), extracellular enzyme activity (B: chitobiase, C: beta-glucosidase; n = 5), and oxygen respiration rates (D; n = 5) of the sediment community in the different treatments under 250 atm *in situ* pressure conditions. Gray bars show the range at the beginning of the incubation. Black dots show measurements after 23 days of incubation, with the horizontal black line indicating the mean value per treatment. Letters indicate groups of treatments that are significantly different from each other based on TukeyHSD at a significance threshold of $p < 0.05$. Sediment treatments: unfed control sediment after 23 days of incubation (unfed), sediments amended with chitin (CHI), *Thalassiosira weissflogii* (TWEI), *Emiliania huxleyi* (EHUX), *Bacillaria* sp. (BCLA) and *Melosira arctica* (MARC). Only one measurement of cell abundance was available for MARC-treated sediment.

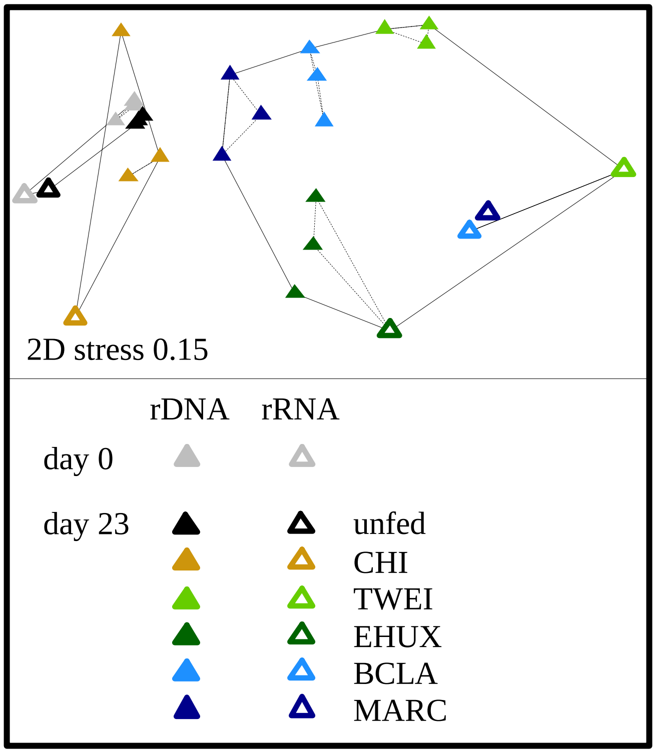

Supplementary Figure S4 Non-metric multidimensional scaling (NMDS) plot based on Bray-Curtis dissimilarity of the total (16S rDNA; n = 3) and active (16S rRNA; n = 1) bacterial community in the different sediment treatments under 250 atm in situ pressure conditions. Hulls displayed by solid lines are based on a dissimilarity threshold of 70%. Hulls displayed by dashed lines are based on a dissimilarity threshold of 32%, and shows clusters of biological replicates. Sediment treatments: unfed control sediment after 23 days of incubation (unfed), sediments amended with chitin (CHI), *Thalassiosira weissflogii* (TWEI), *Emiliania huxleyi* (EHUX), *Bacillaria* sp. (BCLA) and *Melosira arctica* (MARC). Only one measurement of cell abundance was available for MARC-treated sediment. No rRNA data is available for MARC-treated.

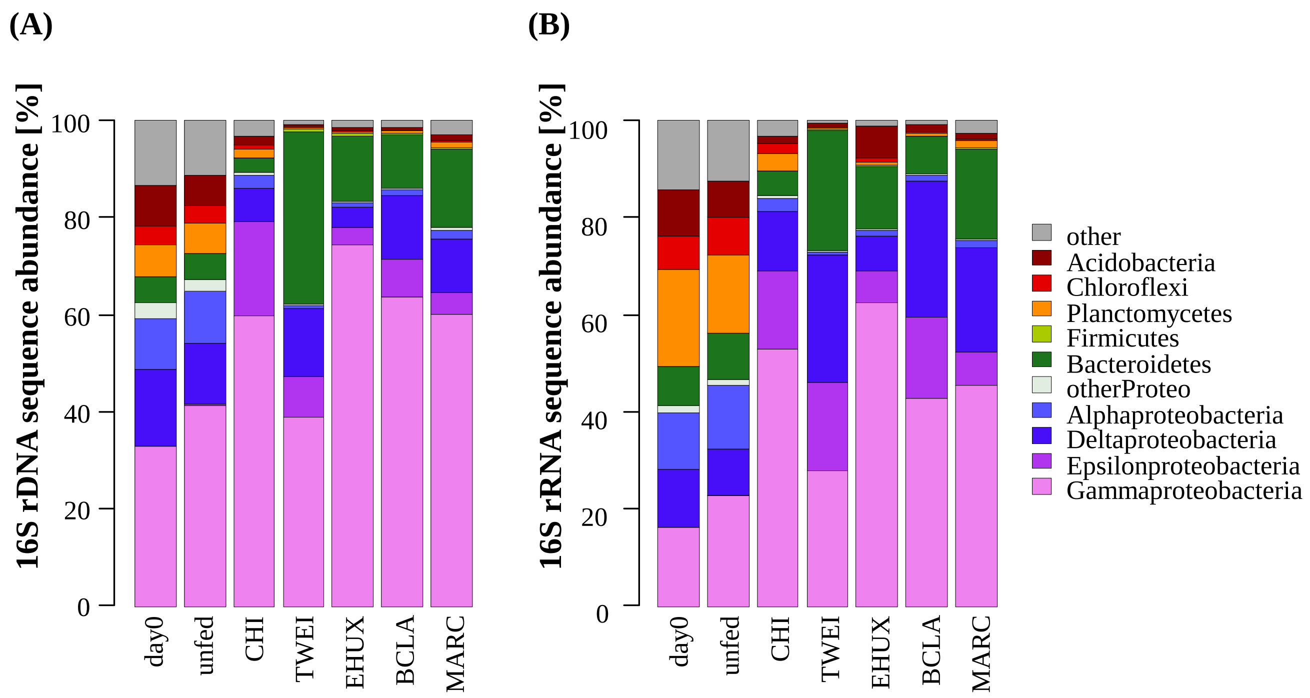

Supplementary Figure S5 Dominant phyla of the total (A: 16S rDNA; n = 3) and active (B: 16S rRNA; n = 1) bacterial community in the different sediment treatments under 250 atm *in situ* pressure conditions. For the total bacterial community, sequences from replicate samples were pooled for the calculation of relative sequence abundances. For *Proteobacteria*, class-level resolution is shown. Sediment treatments: unfed control sediment after 23 days of incubation (unfed), sediments amended with chitin (CHI), *Thalassiosira weissflogii* (TWEI), *Emiliania huxleyi* (EHUX), *Bacillaria* sp. (BCLA) and *Melosira arctica* (MARC). No rRNA data is available for MARC-treated sediment.

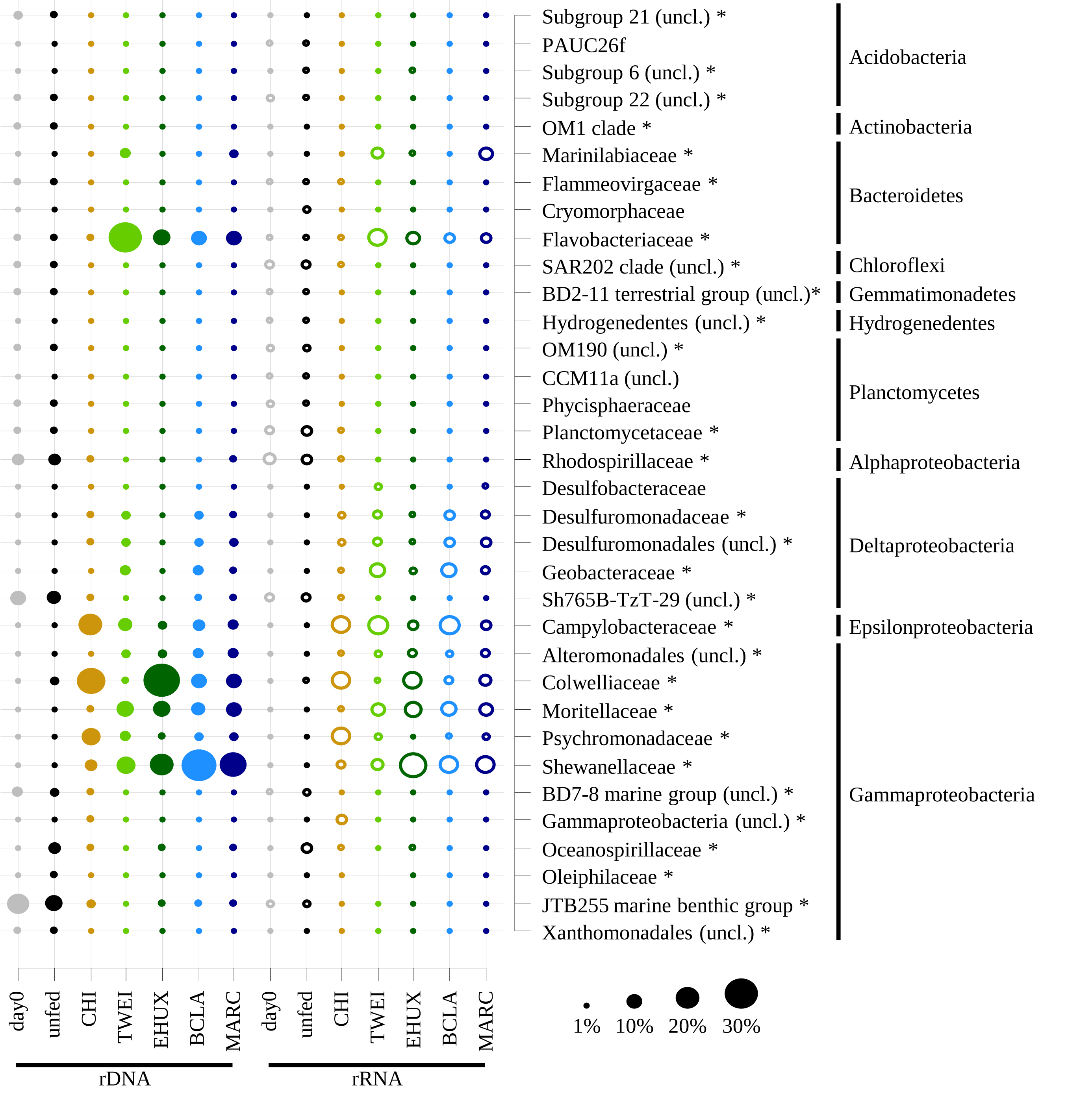

Supplementary Figure S6 Dot plot showing relative sequence abundances of dominant bacterial families, and their phylum affiliation, of the total (16S rDNA; n = 3) and active (16S rRNA; n = 1) bacterial community in the different sediment treatments under 250 atm *in situ* pressure conditions. For the total bacterial community, sequences from replicate samples were pooled for the calculation of relative sequence abundances. For families of the *Proteobacteria*, class-level resolution is shown. For taxa that were unclassified at the respective level of resolution, the next higher taxonomic rank is shown. Asterisks mark differentially abundant taxa between treatments based on 16S rDNA samples (ALDEx2 analysis). All groups that are not marked by an asterisk were only abundant in 16S rRNA data, for which no replicates are available and therefore no analysis of differential abundance could be performed. Sediment treatments: unfed control sediment after 23 days of incubation (unfed), sediments amended with chitin (CHI), *Thalassiosira weissflogii* (TWEI), *Emiliania huxleyi* (EHUX), *Bacillaria* sp. (BCLA) and *Melosira arctica* (MARC). No rRNA data is available for MARC-treated sediment.
